# Associations of fatty acids with the risk of biliary tract calculus and inflammation: a Mendelian randomization study

**DOI:** 10.1186/s12944-023-01989-8

**Published:** 2024-01-08

**Authors:** Xing-Ming Xie, Tao Liu, Guo-Ying Wang

**Affiliations:** 1 Department of Hepatobiliary Surgery, The First Affiliated Hospital of Guangzhou Medical University, Guangzhou, Guangdong 510120 People’s Republic of China; 2 Department of Hepatobiliary Surgery, The Central Hospital of Enshi Tujia and Miao Autonomous Prefecture, Enshi, Hubei 445000 People’s Republic of China

**Keywords:** Mendelian randomization, Risk factor, Fatty acid, Genome-wide association study, Cholecystolithiasis, Bile duct calculus

## Abstract

**Background:**

The presence of gallstones in both the gallbladder and bile ducts is referred to as cholelithiasis. The prevalence of cholecystolithiasis and bile duct stones differs. Observational and Mendelian randomization (MR) studies have elucidated the significant contributing role of numerous fatty acids (FAs) in the development of cholelithiasis. Despite numerous studies about cholelithiasis, evidence on the relationship between serum FA levels and cholecystolithiasis, as well as bile duct stones with or without inflammation, remains insufficient.

**Methods:**

A two-sample MR study was designed to clarify the impact of serum FA levels on various bile duct inflammatory diseases. The summary statistics of single nucleotide polymorphisms (SNPs) associated with fatty acids were obtained from the UK Biobank (UKB) and included data from 114,999 participants. The researchers obtained GWAS summary statistics for cholecystolithiasis and bile duct stones in 463,010 and 361,194 European participants, including cases with and without inflammation. No sample overlap between the exposure and outcome was verified through the “mr-lap” package. The SNPs were screened to identify instrumental variables (IVs). Cochran’s Q test was applied for heterogeneity assessment. Inverse variance weighting (IVW) (fixed effects or random effects), MR-Egger regression and weighted median methods were used for MR. Multivariable MR was applied to determine the direct effect of each exposure on the outcome. A false discovery rate (FDR) was applied to adjust for multiple testing correction based on the Benjamini-Hochberg method. Finally, the FinnGen Consortium was used to validate some results.

**Results:**

The overall concentration of polyunsaturated fatty acids (PUFAs) in the serum was negatively associated with the risk of calculus of the gallbladder with acute cholecystitis (IVW, OR = 0.996, *P* = 0.038, CI 0.992–0.999; weighted median, OR = 0.995, *P* = 0.025, CI 0.991–0.999). The percentage of PUFAs to total monounsaturated fatty acids(MUFAs) (IVW, OR = 0.998, *P* = 0.045, CI 0.997–0.999) and the percentage of PUFAs to total FAs (IVW, OR = 0.997, *P* = 0.025, CI 0.995–0.999) had a protective role against cholecystitis. The percentage of PUFAs to total FAs had a protective role against calculus of the gallbladder without cholecystitis (IVW, OR = 0.995, *P* = 0.026, CI 0.990–0.999; MR Egger, OR = 0.99, *P* = 0.03, CI 0.982–0.998; weighted median, OR = 0.991, *P* = 5.41e-06, CI 0.988–0.995). Conversely, the percentage of MUFAs to total FAs increased the risk for cholecystitis (IVW, OR = 1.001, *P* = 0.034, CI 1.0001–1.002). However, there were no causal effects of the above exposures on the outcomes through multivariable MR and multiple testing correction. Finally, the causal effects of the above exposures on cholecystitis were validated in the FinnGen Consortium, which suggested that the percentage of PUFAs to total FAs (IVW, OR = 0.744, *P* = 0.021, CI 0.579–0.957) had a protective role against cholecystitis.

**Conclusion:**

These Mendelian randomization findings suggested that more attention should be focused on people who have low serum PUFA levels, which may have a potential role in the occurrence of calculus of the gallbladder or cholecystitis rather than calculus of the bile duct without cholangitis or cholecystitis.

**Supplementary Information:**

The online version contains supplementary material available at 10.1186/s12944-023-01989-8.

## Introduction

Cholelithiasis consists of cholecystolithiasis and bile duct stones with or without corresponding inflammation. The symptoms and outcomes caused by cholecystolithiasis and bile duct stones differ greatly. Cholecystolithiasis can induce cholecystitis or gallbladder cancer and affects liver function less than bile duct stones. However, bile duct stones have more pernicious effects on liver function, and it is more challenging to completely clear the hepatoliths. The incidence of cholecystolithiasis and bile duct stones also differs [1–4]. Abnormalities in FAs and cholesterol metabolism significantly contribute to pathological processes [5–7]. Studies have explored the causal effects of some kinds of fatty acids on cholelithiasis. Low serum PUFA levels contribute to an elevated risk of cholelithiasis [8, 9]. The proportion of omega-6 PUFAs compared to omega-3 PUFAs is positively associated with cholelithiasis, and total omega-3 PUFA and docosahexaenoic acid (DHA) levels are both negatively associated with cholelithiasis [10]. In a C57BL/6J mouse lithogenic diet model, eicosapentaenoic acid (EPA) and DHA had the ability to inhibit gallstone formation [11]. Cholelithiasis is a general term for diseases in which stones occur in any part of the biliary system (including the gallbladder and bile ducts) with or without inflammation. However, there are no studies that focus the causal links between fatty acids and sub-classification of cholelithiasis, which includes cholecystolithiasis and bile duct stones with or without corresponding inflammation. The evidence regarding the potential causal links between fatty acids and cholecystolithiasis, as well as bile duct stones with or without inflammation, remains insufficient.

MR is a developing method that employs gene variants as IVs to evaluate the causal role of an exposure in the development of an outcome. The MR method is more advanced than traditional observational studies due to its remarkable capacity to significantly minimize bias arising from confounding factors and reverse causality, as has been previously documented in studies [12–14]. Here, an experimental design was developed to identify the underlying causes of the impact of circulating MUFAs, PUFAs, and saturated fatty acid(SFAs), the percentage of MUFAs to total FAs, the percentage of PUFAs to total MUFAs, the percentage of PUFAs to total FAs, and the percentage of SFAs to total FAs on cholecystolithiasis and bile duct stones with or without corresponding inflammation based on genome-wide association study (GWAS) datasets and the FinnGen Consortium [15].

## Materials and methods

This study adhered to the Strengthening the Reporting of Observational Studies in Epidemiology Using MR rules [16].

### Data sources

The data regarding exposure and outcome sources were obtained from the “openGWAS” project. The serum total MUFAs (study ID “met-d-MUFA”), PUFAs (study ID “met-d-PUFA”), the percentage of MUFAs to total FAs (study ID “met-d-MUFA_pct”), the percentage of PUFAs to MUFAs (study ID “met-d-PUFA_by_MUFA”), the percentage of PUFAs to total FAs (study ID “met-d-PUFA_pct”), the percentage of SFAs to total FAs (study ID “met-d-SFA_pct”), and FAs (study ID “met-d-SFA”) served as the exposure sources from the European population, including 114,999 participants from the MRCIEU OpenGWAS project [17], for whom the data were adjusted for age, age squared, and sex. The fatty acid concentration was determined by a targeted high-throughput nuclear magnetic resonance metabolomics platform (Nightingale Health Ltd; biomarker quantification version 2020), and 121,577 samples were included in the fatty acid examination [17].

The UKB outcomes were defined according to International Classification of Diseases-10th revision (ICD-10) codes, including calculus of the bile duct without cholangitis or cholecystitis (1,706 cases, 461,304 controls; study ID “ukb-b-8268”; ICD 10 code: K80.5), calculus of the gallbladder without cholecystitis (5,766 cases, 457,244 controls; study ID “ukb-b-11020”, ICD 10 code: K80.2), cholecystitis (1,930 cases, 359,264 controls; study ID “ukb-d-K81”, ICD 10 code: K81), and calculus of the gallbladder with acute cholecystitis (1,100 cases, 461,910 controls; study ID “ukb-b-10362”, ICD 10 code: K80.0) for the European population. A cohort of cholecystitis patients (4,299 cases, 330,903 controls; ICD 10 code: K81) from the FinnGen Consortium (https://r9.finngen.fi/) was included.

The MRlap package was employed to determine the sample overlap between the exposure and outcome GWASs [18], and no sample overlap was verified in the present study.

### Mendelian randomization design

**Fig. 1:**
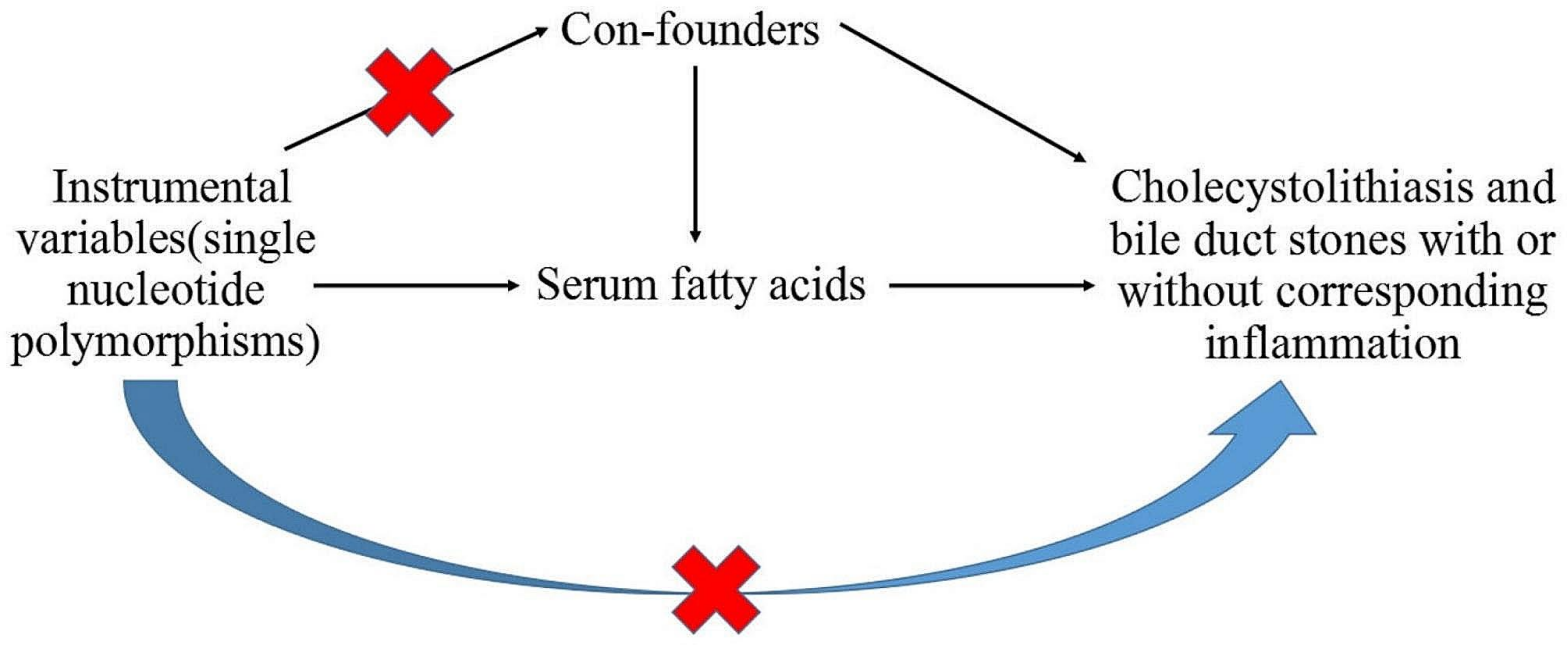
Basic assumptions of mendelian randomization

The MR study complied with three principal assumptions (Fig. 1): (1) the relevance assumption, stating that the instrumental variables (IVs) are highly correlated with the exposure; (2) the assumption of exchangeability, stating that the independent variables (IVs) are not linked to any potential confounders; and (3) the assumption of the exclusion restriction, stating that the instrumental variables (IVs) are solely associated with the exposure and are not directly associated with the outcome [19]. The SNPs serving as instrumental variables were carefully selected based on their remarkable genome-wide differences (*P* < 5*10^–8^) with linkage disequilibrium (LD) and the genome region (R^2^ < 0.001 within 10,000 kilobases). To avoid weak IVs, IVs were removed if their F statistics were determined to be < 10 using the following formula: F = beta^2^/se^2^, where “beta” is the effect size of the IV on the exposure, and “se” is the corresponding standard error [20, 21]. To test assumption 3, we used PhenoScanner _v2_ for detecting potential confounders among the IVs. Confounders were defined as factors that could have an important effect on the occurrence of cholelithiasis. The confounders were total cholesterol, triglycerides, HDL cholesterol, type 2 diabetes, low-density lipoprotein, body mass index, chronic hepatitis C virus infection, liver cirrhosis, alcohol and smoking.

### Two-sample Mendelian randomization analysis

The inverse variance weighting (IVW) (fixed effects or random effects if heterogeneity existed), MR-Egger regression and weighted median methods were used for the MR analysis. The causal effects of the exposures, including total MUFAs, PUFAs, SFAs, the percentage of MUFAs to total FAs, the percentage of PUFAs to total MUFAs, the percentage of PUFAs to total FAs, and the percentage of SFAs to total FAs, on each outcome, such as calculus of the bile duct without cholangitis or cholecystitis, calculus of the gallbladder without cholecystitis, cholecystitis, and calculus of the gallbladder with acute cholecystitis, were tested using the MR method. For the examination of heterogeneity, Cochran’s Q test was applied [22], and *P* < 0.05 was considered to indicate significant heterogeneity among the IVs. Moreover, horizontal pleiotropy was evaluated using an MR-Egger intercept method [23] and MR-Pleiotropy RESidual Sum and Outlier (PRESSO) [24]. Horizontal pleiotropy was considered absent if the intercept was close to 0 (*P* > 0.05), and if outliers were detected by MR-PRESSO analysis, MR causal estimation was reassessed after the outliers were removed. For the power calculation, the power to detect an OR of 0.90 or 1.10 was determined using an online tool with a type-1 error rate of 0.05 (http://cnsgenomics.com/shiny/mRnd/).

### Multivariable Mendelian randomization

Multivariable MR was used to estimate the direct effect of each exposure on the outcome, that is, an effect that was not mediated by any other fatty acid in the model.

### Statistical software

The “Two-sample MR” and “MendelianRandomization” packages were used in this study using R programming language, specifically version 4.3.1. The “MRPRESSO” package performed the MR-PRESSO analysis, and the “MVMR” package performed the multivariable Mendelian randomization analysis. A significant difference was considered when the *P* value was less than 0.05.

## Results

Potential cofounders were excluded using PhenoScanner _v2_. Ultimately, 20 IVs(serum total MUFAs), 26 IVs(serum total PUFAs), 43 IVs(the percentage of MUFAs to total FAs), 37 IVs(the percentage of PUFAs to total MUFAs), 30 IVs(the percentage of PUFAs to total FAs), 14 IVs(the percentage of SFAs to total FAs) and 23 IVs(SFAs) were enrolled in the MR analysis, respectively, for which all IVs F statistics were not less than 10 (Supplementary S1).

### Mendelian randomization

#### Impact of serum FA levels on biliary tract calculus and inflammation

The total PUFAs decreased the risk of calculus of the gallbladder with acute cholecystitis (IVW, OR = 0.996, *P* = 0.038, CI 0.992–0.999; weighted median, OR = 0.995, *P* = 0.025, CI 0.991–0.999) (Table 1).

**Table 1:** Causal effect of polyunsaturated fatty acids on biliary tract calculus and inflammation

Exposure	Outcome	MR method	Number of SNP	OR (95% CI)	*P* value	Test of heterogeneity		Test of pleiotropy	
						Cochrane Q test	*P* of heterogeneity	MR-Egger intercept	*P* of pleiotropy
Polyunsaturated fatty acids	Calculus of bile duct without cholangitis or cholecystitis	Inverse variance weighted	9	1.002(0.998–1.007)	0.161	16.013	0.042		
		MR Egger		1.016(0.998–1.034)	0.114	12.079	0.097	-0.0004	0.174
		weighted median		1.002(0.997–1.006)	0.327				
	Calculus of gallbladder without cholecystitis	Inverse variance weighted	15	1.003(0.997–1.009)	0.239	35.553	0.001		
		MR Egger		1.000(0.980–1.020)	0.997	35.168	0.0007	0.0001	0.712
		weighted median		1.000(0.994–1.007)	0.789				
	Cholecystitis	Inverse variance weighted	26	1.001(0.998–1.003)	0.418	32.666	0.139		
		MR Egger		1.001(0.995–1.006)	0.702	32.666	0.111	-1.368374e-06	0.990
		weighted median		1.007(0.997–1.003)	0.639				
	Calculus of gallbladder with acute cholecystitis	Inverse variance weighted	6	0.996(0.992–0.999)	0.038	6.849	0.232		
		MR Egger		1.003(0.972–1.036)	0.818	6.476	0.166	-0.0002	0.656
		weighted median		0.995(0.991–0.999)	0.025				

The percentage of PUFAs to total MUFAs (IVW, OR = 0.998, *P* = 0.045, CI 0.997–0.999) (Table S2) and the percentage of PUFAs to total FAs (IVW, OR = 0.997, *P* = 0.025, CI 0.995–0.999) had a protective role against cholecystitis (Table S3).

The percentage of PUFAs to total FAs (IVW, OR = 0.995, *P* = 0.026, CI 0.990–0.999; MR Egger, OR = 0.99, *P* = 0.03, CI 0.982–0.998; weighted median, OR = 0.991, *P* = 5.41e-06, CI 0.988–0.995) had a protective role against calculus of the gallbladder without cholecystitis (Table S3).

The percentage of MUFAs to total FAs increased the risk for cholecystitis (IVW, OR = 1.001, *P* = 0.034, CI 1.0001–1.002) (Table S4). For the power calculation, the power to detect an OR of 0.90 or 1.10 is listed in Table 2.

**Table 2:** Power calculation: significant phenotypes for outcomes

Significant phenotypes for outcomes	Sample	VE per IV	Case proportion	Power
PUFA for calculus of gallbladder with acute cholecystitis	463,010	0.23%	0.24%	2.50%
percentage of MUFAs to total FAs for cholecystitis	361,194	4.10%	0.53%	2.60%
percentage of PUFAs to total FAs for Calculus of gallbladder without cholecystitis	463,010	1.69%	1.25%	2.80%
Percentage of PUFAs to total FAs for cholecystitis	361,194	1.92%	0.53%	2.60%
Percentage of PUFAs to total MUFAs for cholecystitis	361,194	3.59%	0.53%	2.60%

Note: Based on two-sided α = 0.05. Abbreviations: VE per IV, variation explained per instrumental variable

The MR analysis indicated that serum total MUFAs, the percentage of SFAs to total FAs, and SFAs had no causal effects on the risk of calculus of the bile duct without cholangitis or cholecystitis, calculus of the gallbladder without cholecystitis, cholecystitis, or calculus of the gallbladder with acute cholecystitis (Tables S5, S6 and S7).

### Multiple testing correction

Due to the high number of exposures and outcomes, a false discovery rate (FDR) was applied to adjust for multiple testing correction based on the Benjamini-Hochberg method [25], and the adjusted *P* values of causal effects of exposures on outcomes exceeded 0.05 (Table S8).

### Multivariable mendelian randomization (MVMR) analysis

When PUFAs, MUFAs and SFAs were simultaneously enrolled into MVMR analysis based on the IVW model and the MR-Egger method, no significant results were observed (Supplementary S2); As additionally, no significant results were observed in the MVMR analysis when the percentage of SFAs to total FAs, the percentage of PUFAs to total FAs, and the percentage of MUFAs to total FAs were assessed together (Supplementary S3).

### Summary of sensitivity analyses

No statistically significant pleiotropic effects verified by the MR-Egger intercept method were observed among the IVs. Funnel plots were generated and displayed a symmetrical distribution of the IVs (Supplementary Fig. 2). The leave-one-out analyses also demonstrated that the causal association was not remarkably influenced by specific SNPs (Supplementary Fig. 3).

### Validation of some causal associations with the FinnGen Consortium

In the FinnGen Consortium, causal associations of exposures with cholecystitis were explored, and the results showed that the percentage of PUFAs to total FAs (IVW, OR = 0.744, *P* = 0.021, CI 0.579–0.957) had a protective role against cholecystitis (Supplementary Table S9).

## Discussion

Extensive research has been conducted on the correlation between fatty acids and the development of cholelithiasis. However, the uncertain relationship between fatty acids and cholecystolithiasis as well as bile duct stones with or without corresponding inflammation is still unclear, and this was the focus of this MR analysis.

Due to hepatic cholesterol hypersecretion, gallbladder hypomotility and supersaturated bile juice, cholesterol crystallization occurs in bile juice, and biliary stones are eventually generated [26]. In this MR study, serum total MUFA levels had no effects on the risk of calculus of the bile duct without cholangitis or cholecystitis, calculus of the gallbladder without cholecystitis, cholecystitis, or calculus of the gallbladder with acute cholecystitis. In a clinical trial, the consumption of high-MUFA diets had the ability to reduce plasma cholesterol concentrations, however, the enrolled cohort was small (n = 22) [27]. Yoshinaga et al. [28] reported that the MUFAs cis-5-eicosenoic acid and cis-7-eicosenoic acid combined with n-3-type PUFAs (EPA/DHA) significantly decreased total cholesterol in HepG2 cells. It is possible that n-3 type PUFAs exert an opposite function in cholesterol generation than cis-5-eicosenoic acid and cis-7-eicosenoic acid, as the present MR findings showed that PUFAs had a potential negative relation with bile duct stones. In addition, Compagnucci et al. [29] reported that consuming large amounts of MUFAs was a risk factor for cholecystolithiasis. On the other hand, it was reported that increased gallbladder prostaglandin I_2_ and prostaglandin E_2_ (PGE_2_) synthesis were related to human cholecystitis [30]. After nonalcoholic fatty liver disease patients received fibre supplementation, their serum total MUFA levels increased with a reduction in PGE_2_ [31], however, only 28 Caucasian participants were enrolled, which means that the cohort was small and that the results hardly supported that total MUFA levels or other fatty acids from fibre intake had direct causal effects on PGE_2_. In the current MR analysis, a high percentage of MUFAs to total FAs was a potential risk factor for cholecystitis, which suggests that high MUFA levels may be a risk factor for cholecystitis. Controversy still exists regarding the causal effects of serum total MUFA levels on cholelithiasis. More mechanical exploration and large-scale cohort studies should be performed to resolve the above questions.

Univariable MR analysis showed that a high serum total PUFA level, a high percentage of PUFAs to total MUFAs and a high percentage of PUFAs to total FAs had a protective role against calculus of the gallbladder with acute cholecystitis or cholecystitis or calculus of the gallbladder without acute cholecystitis. However, the above exposures did not show a protective role in multivariable MR analyses or after multiple testing correction. There are inevitable inner relationships among FAs, that is, the FAs can convert into each other, which may account for negative results in multivariable MR analyses. However, a protective effect of the percentage of PUFAs to total FAs against cholecystitis was verified in the FinnGen Consortium. Together, these findings also suggest that PUFAs may have a protective role against cholecystitis or calculus of the gallbladder.

In a previous observational study, PUFA intake was not related to cholecystolithiasis [29]. In contrast, researchers reported that PUFA intake was found to have an adverse association with the occurrence of gallstone disease, specifically in males [32]. In a cholecystitis model in cats, a significant elevation in PGE_2_ was found [33]. In cell experiments, DHA inhibited hepatocellular carcinoma cell growth via inhibition of the PGE_2_ signalling pathway [34]. Thus, DHA, a kind of omega-3 PUFA, may exert a protective role against cholecystitis through the PGE_2_ signalling pathway. In addition, a reduction in high-density lipoprotein (HDL) cholesterol and an increase in triglycerides contribute to the development of cholelithiasis. It was reported that omega-6 PUFAs may significantly increase HDL cholesterol levels and decrease triglyceride levels [35]. PUFAs mainly include two categories: omega-3/6 PUFAs. Therefore, the total PUFA levels displayed a potential protective role against cholecystitis and calculus of the gallbladder with acute cholecystitis in this MR study.

In the bile secreted from hepatic ducts that contain stones, the levels of phospholipase A2 (sPLA2) were found to be considerably elevated compared to the bile in the ducts from patients with gallbladder stones. The heightened sPLA2 level was linked to a simultaneous rise in PGE_2_ [36]. Enhanced synthesis of PGE2 resulting from sPLA2-/COX2 in hepatolithiasis was observed, potentially implicating PGE_2_ in the development of chronic proliferative cholangitis [37]. As mentioned earlier, some specified MUFAs and PUFAs can inhibit the PGE_2_ signalling pathway, which suggests their protective role against calculus of the bile duct. In our MR analysis, the total MUFAs or PUFAs level did not display causal effects on calculus of the bile duct, which indicates that more mechanistic or large-scale observational studies need to be conducted.

### Study strengths and limitations

This research had numerous advantages. First, a large sample from the GWAS database was employed, which enhances the reliability of the conclusions. Second, cholelithiasis has a high morbidity rate, and many studies have focused on it, however, cholelithiasis comprises cholecystolithiasis and bile duct stones with or without corresponding inflammation, which have different symptoms and regimens. In this study, the causal impacts of FAs on the development of different kinds of bile duct diseases were studied, which will help people to instruct themselves to adjust food to maintain health with more useful information. However, this study had several limitations. First, the results were obtained from the data of European populations, which were cautiously used for other populations, such as Asians or Africans. Second, this MR analysis showed a linear relationship between the exposures and outcomes in causal analysis, and these causal effects may be weakened by other potential nonlinear relationships, such as J- or U-shaped relationships. Third, the researchers adopted the MR-Egger intercept method to verify the potential pleiotropy, and if the outliers were detected by MR-PRESSO, MR causal estimation was reassessed after the outliers were removed. However, the potential pleiotropy could not be removed completely.

## Conclusion

These Mendelian randomization findings suggested that more attention should be focused on people who have low serum PUFA levels, which may have a potential role in the occurrence of calculus of the gallbladder or cholecystitis rather than calculus of the bile duct without cholangitis or cholecystitis.

### Electronic supplementary material

Below is the link to the electronic supplementary material.

**Supplementary S1:** The F statistics of IVs of serum total MUFAs, serum total PUFAs, percentage of MUFAs to total FAs, percentage of PUFAs to total MUFAs, percentage of PUFAs to total FAs, percentage of SFAs to total FAs, and SFAs, respectively.

**Supplementary S2:** MVMR analysis(PUFAs, MUFAs and SFAs) based on IVW model and the MR-Egger method.

**Supplementary S3:** MVMR analysis(percentage of SFAs to total FAs, percentage of PUFAs to total FAs, percentage of MUFAs to total FAs) based on IVW model and the MR-Egger method.

**Supplementary Tables S2–S9:** **Table S2.** Causal effect of percentage of polyunsaturated fatty acids to monounsaturated fatty acids on biliary tract calculus and inflammation. **Table S3.** Causal effect of percentage of polyunsaturated fatty acids to total fatty acids on biliary tract calculus and inflammation. **Table S4.** Causal effect of percentage of monounsaturated fatty acids to total fatty acids on biliary tract calculus and inflammation. **Table S5.** Causal effect of monounsaturated fatty acids on biliary tract calculus and inflammation. **Table S6.** Causal effect of percentage of saturated fatty acids to total fatty acids on biliary tract calculus and inflammation. **Table S7.** Causal effect of saturated fatty acids on biliary tract calculus and inflammation. **Table S8.** Multiple testing correction: the adjusted P values of causal effects of exposures on outcomes. **Table S9.** Causal effects of fatty acids on cholecystitis based on FinnGen Consortium.

**Supplementary Figure2:** Funnel plots of the enrolled SNPs. (A1-A4) MUFA-related SNPs, (B1-B4) Percentage of MUFA to total FAs-related SNPs, (C1-C4) Percentage of PUFA to total FAs-related SNPs, (E1-E4) PUFA-related SNPs, (F1-F4) Percentage of PUFA to MUFA-related SNPs, (G1-G4) SFA-related SNPs in calculus of bile duct without cholangitis or cholecystitis, calculus of gallbladder with acute cholecystitis, calculus of gallbladder without cholecystitis, and cholecystitis, respectively. (D1-D3) Percentage of SFA to total FAs-related SNPs in calculus of bile duct without cholangitis or cholecystitis, calculus of gallbladder without cholecystitis, and cholecystitis, respectively.

**Supplementary Figure3:** The leave-one-out analysis. (A1-A4) MUFA-related SNPs, (B1-B4) Percentage of MUFA to total FAs-related SNPs, (C1-C4) Percentage of PUFA to total FAs-related SNPs, (E1-E4) PUFA-related SNPs, (F1-F4) Percentage of PUFA to MUFA-related SNPs, (G1-G4) SFA-related SNPs in calculus of bile duct without cholangitis or cholecystitis, calculus of gallbladder with acute cholecystitis, calculus of gallbladder without cholecystitis, and cholecystitis, respectively. (D1-D3) Percentage of SFA to total FAs-related SNPs in calculus of bile duct without cholangitis or cholecystitis, calculus of gallbladder without cholecystitis, and cholecystitis, respectively.

## Data Availability

The data generated and/or analyzed are available from the corresponding author on reasonable request.

## Abbreviations

DHA: Docosahexaenoic acid
EPA: Eicosapentaenoic acid
FA: Fatty acid
GWAS: Genome-wide association study
HDL: High-density lipoprotein
IVs: Instrumental variables
IVW: Inverse variance weighting
LD: Linkage disequilibrium
MR: Mendelian randomization
MUFA: Monounsaturated fatty acids
PGE_2_: Prostaglandin E_2_
PRESSO: Pleiotropy RESidual Sum and Outlier
PUFA: Polyunsaturated fatty acids
SFA: Saturated fatty acid
SNPs: Single nucleotide polymorphisms
sPLA2: Secreted phospholipases A2
UKB: UK Biobank
