# Supplementary material for: Associations of fatty acids with the risk of biliary tract calculus and inflammation: a Mendelian randomization study
Source: Lipids Health Dis. 2024 Jan 8;23:8. doi: 10.1186/s12944-023-01989-8 (PMC10773125; doi:10.1186/s12944-023-01989-8)
Supplement: Supplementary file 4 — Supplementary Tables S2–S9: Table S2. Causal effect of percentage of polyunsaturated fatty acids to monounsaturated fatty acids on biliary tract calculus and inflammation. Table S3. Causal effect of percentage of polyunsaturated fatty acids to total fatty acids on biliary tract calculus and inflammation. Table S4. Causal effect of percentage of monounsaturated fatty acids to total fatty acids on biliary tract calculus and inflammation. Table S5. Causal effect of monounsaturated fatty acids on biliary tract calculus and inflammation. Table S6. Causal effect of percentage of saturated fatty acids to total fatty acids on biliary tract calculus and inflammation. Table S7. Causal effect of saturated fatty acids on biliary tract calculus and inflammation. Table S8. Multiple testing correction: the adjusted P values of causal effects of exposures on outcomes. Table S9. Causal effects of fatty acids on cholecystitis based on FinnGen Consortium. [file 12944_2023_1989_MOESM4_ESM.doc]

Table S2 Causal effect of percentage of polyunsaturated fatty acids to monounsaturated fatty acids on biliary tract calculus and inflammation

| Exposure | Outcome | MR method | Number of SNP | OR (95% CI) | *P* value | Test of heterogeneity | |  | Test of pleiotropy | |
| --- | --- | --- | --- | --- | --- | --- | --- | --- | --- | --- |
|  |  |  |  |  |  | Cochrane Q test | *P* of  heterogeneity |  | MR-Egger intercept | *P* of  pleiotropy |
| percentage of polyunsaturated fatty acids to monounsaturated fatty acids | Calculus of bile duct without cholangitis or cholecystitis | Inverse variance weighted | 17 | 0.999(0.997-1.000) | 0.153 | 18.458 | 0.297 |  |  |  |
|  |  | MR Egger |  | 0.999(0.997-1.001) | 0.611 | 18.114 | 0.256 |  | -2.954e-05 | 0.601 |
|  |  | weighted median |  | 0.999(0.997-1.000) | 0.322 |  |  |  |  |  |
|  | Calculus of gallbladder without cholecystitis | Inverse variance weighted | 23 | 0.997(0.993-1.000) | 0.091 | 52.750 | 0.0006 |  |  |  |
|  |  | MR Egger |  | 1.004(0.996-1.011) | 0.276 | 52.482 | 0.0004 |  | 4.431e-05 | 0.734 |
|  |  | weighted median |  | 0.997(0.992-1.001) | 0.251 |  |  |  |  |  |
|  | Cholecystitis | Inverse variance weighted | 36 | 0.998(0.997-0.999) | 0.045 | 39.384 | 0.280 |  |  |  |
|  |  | MR Egger |  | 0.998(0.996-1.001) | 0.416 | 39.101 | 0.251 |  | -3.237e-05 | 0.622 |
|  |  | weighted median |  | 0.998(0.996-1.000) | 0.080 |  |  |  |  |  |
|  | Calculus of gallbladder with acute cholecystitis | Inverse variance weighted | 8 | 0.999(0.997-1.000) | 0.473 | 13.830 | 0.054 |  |  |  |
|  |  | MR Egger |  | 0.998(0.996-1.000) | 0.156 | 10.013 | 0.124 |  | 0.0001 | 0.181 |
|  |  | weighted median |  | 0.999(0.997-1.000) | 0.102 |  |  |  |  |  |

Table S3 Causal effect of percentage of polyunsaturated fatty acids to total fatty acids on biliary tract calculus and inflammation

| Exposure | Outcome | MR method | Number of SNP | OR (95% CI) | *P* value | Test of heterogeneity | |  | Test of pleiotropy | |
| --- | --- | --- | --- | --- | --- | --- | --- | --- | --- | --- |
|  |  |  |  |  |  | Cochrane Q test | *P* of  heterogeneity |  | MR-Egger intercept | *P* of  pleiotropy |
| percentage of polyunsaturated fatty acids to total fatty acids | Calculus of bile duct without cholangitis or cholecystitis | Inverse variance weighted | 14 | 0.998(0.996-1.000) | 0.234 | 17.323 | 0.184 |  |  |  |
|  |  | MR Egger |  | 0.998(0.995-1.002) | 0.569 | 17.311 | 0.138 |  | -6.951e-06 | 0.929 |
|  |  | weighted median |  | 0.998(0.996-1.000) | 0.288 |  |  |  |  |  |
|  | Calculus of gallbladder without cholecystitis | Inverse variance weighted | 19 | 0.995(0.990-0.999) | 0.026 | 45.524 | 0.0003 |  |  |  |
|  |  | MR Egger |  | 0.990(0.982-0.998) | 0.030 | 41.093 | 0.0009 |  | 0.0002 | 0.193 |
|  |  | weighted median |  | 0.991(0.988-0.995) | 5.41e-06 |  |  |  |  |  |
|  | Cholecystitis | Inverse variance weighted | 27 | 0.997(0.995-0.999) | 0.025 | 25.465 | 0.492 |  |  |  |
|  |  | MR Egger |  | 0.997(0.993-1.000) | 0.131 | 25.292 | 0.446 |  | 3.239e-05 | 0.682 |
|  |  | weighted median |  | 0.997(0.994-1.000) | 0.078 |  |  |  |  |  |
|  | Calculus of gallbladder with acute cholecystitis | Inverse variance weighted | 6 | 0.999(0.997-1.001) | 0.354 | 8.205 | 0.145 |  |  |  |
|  |  | MR Egger |  | 0.997(0.994-1.000) | 0.142 | 5.240 | 0.263 |  | 0.0001 | 0.206 |
|  |  | weighted median |  | 0.998(0.997-1.000) | 0.102 |  |  |  |  |  |

Table S4 Causal effect of percentage of monounsaturated fatty acids to total fatty acids on biliary tract calculus and inflammation

| Exposure | Outcome | MR method | Number of SNP | OR (95% CI) | *P* value | Test of heterogeneity | |  | Test of pleiotropy | |
| --- | --- | --- | --- | --- | --- | --- | --- | --- | --- | --- |
|  |  |  |  |  |  | Cochrane Q test | *P* of  heterogeneity |  | MR-Egger intercept | *P* of  pleiotropy |
| percentage of monounsaturated fatty acids to total fatty acids | Calculus of bile duct without cholangitis or cholecystitis | Inverse variance weighted | 11 | 1.000(0.999-1.001) | 0.675 | 11.171 | 0.344 |  |  |  |
|  |  | MR Egger |  | 1.001(0.999-1.002) | 0.114 | 6.766 | 0.661 |  | -0.0001 | 0.065 |
|  |  | weighted median |  | 1.000(0.999-1.001) | 0.438 |  |  |  |  |  |
|  | Calculus of gallbladder without cholecystitis | Inverse variance weighted | 20 | 1.002(0.997-1.006) | 0.298 | 29.489 | 0.058 |  |  |  |
|  |  | MR Egger |  | 1.001(0.989-1.014) | 0.806 | 29.464 | 0.042 |  | 2.811e-05 | 0.902 |
|  |  | weighted median |  | 1.002(0.997-1.008) | 0.294 |  |  |  |  |  |
|  | Cholecystitis | Inverse variance weighted | 39 | 1.001(1.0001-1.002) | 0.034 | 47.709 | 0.134 |  |  |  |
|  |  | MR Egger |  | 1.001(0.999-1.003) | 0.149 | 47.673 | 0.112 |  | -1.103e-05 | 0.868 |
|  |  | weighted median |  | 1.005(0.999-1.003) | 0.077 |  |  |  |  |  |
|  | Calculus of gallbladder with acute cholecystitis | Inverse variance weighted | 5 | 1.0007(0.999-1.001) | 0.133 | 1.510 | 0.824 |  |  |  |
|  |  | MR Egger |  | 1.001(0.999-1.002) | 0.162 | 0.302 | 0.959 |  | -6.936e-05 | 0.352 |
|  |  | weighted median |  | 1.000(0.999-1.001) | 0.091 |  |  |  |  |  |

Table S5 Causal effect of monounsaturated fatty acids on biliary tract calculus and inflammation

| Exposure | Outcome | MR method | Number of SNP | OR (95% CI) | *P* value | Test of heterogeneity | |  | Test of pleiotropy | |
| --- | --- | --- | --- | --- | --- | --- | --- | --- | --- | --- |
|  |  |  |  |  |  | Cochrane Q test | *P* of  heterogeneity |  | MR-Egger intercept | *P* of  pleiotropy |
| Monounsaturated fatty acids | Calculus of bile duct without cholangitis or cholecystitis | Inverse variance weighted | 8 | 1.001(0.997-1.006) | 0.418 | 14.30742 | 0.045 |  |  |  |
|  |  | MR Egger |  | 1.011(0.992-1.031) | 0.277 | 12.141 | 0.058 |  | -0.0002 | 0.34 |
|  |  | weighted median |  | 1.003(0.999-1.007) | 0.097 |  |  |  |  |  |
|  | Calculus of gallbladder without cholecystitis | Inverse variance weighted | 13 | 1.004(0.999-1.009) | 0.059 | 14.307 | 0.045 |  |  |  |
|  |  | MR Egger |  | 0.998(0.979-1.017) | 0.277 | 12.14 | 0.058 |  | -0.0002 | 0.34 |
|  |  | weighted median |  | 1.004(0.997-1.01) | 0.225 |  |  |  |  |  |
|  | Cholecystitis | Inverse variance weighted | 20 | 1.001(0.998-1.004) | 0.278 | 23.75 | 0.205 |  |  |  |
|  |  | MR Egger |  | 1.002(0.996-1.008) | 0.446 | 23.629 | 0.167 |  | -3.81298e-05 | 0.758 |
|  |  | weighted median |  | 1.003(0.999-1.007) | 0.0504 |  |  |  |  |  |
|  | Calculus of gallbladder with acute cholecystitis | Inverse variance weighted | 5 | 1.002(0.998-1.005) | 0.266 | 2.275 | 0.685 |  |  |  |
|  |  | MR Egger |  | 0.993(0.962-1.024) | 0.705 | 1.976 | 0.577 |  | 0.0002 | 0.622 |
|  |  | weighted median |  | 1.002(0.997-1.006) | 0.362 |  |  |  |  |  |

| Exposure | Outcome | MR method | Number of SNP | OR (95% CI) | *P* value | Test of heterogeneity | |  | Test of pleiotropy | |
| --- | --- | --- | --- | --- | --- | --- | --- | --- | --- | --- |
|  |  |  |  |  |  | Cochrane Q test | *P* of  heterogeneity |  | MR-Egger intercept | *P* of  pleiotropy |
| percentage of saturated fatty acids to total fatty acids | Calculus of bile duct without cholangitis or cholecystitis | Inverse variance weighted | 3 | 1.000(0.994-1.005) | 0.918 | 2.007 | 0.366 |  |  |  |
|  |  | MR Egger |  | 1.005(0.970-1.041) | 0.810 | 1.844 | 0.174 |  | -0.0001 | 0.815 |
|  |  | weighted median |  | 1.000(0.994-1.007) | 0.842 |  |  |  |  |  |
|  | Calculus of gallbladder without cholecystitis | Inverse variance weighted | 8 | 0.995(0.988-1.003) | 0.312 | 13.069 | 0.070 |  |  |  |
|  |  | MR Egger |  | 0.993(0.962-1.025) | 0.715 | 13.029 | 0.042 |  | 7.671e-05 | 0.896 |
|  |  | weighted median |  | 0.993(0.985-1.002) | 0.176 |  |  |  |  |  |
|  | Cholecystitis | Inverse variance weighted | 12 | 0.997(0.993-1.001) | 0.263 | 15.729 | 0.151 |  |  |  |
|  |  | MR Egger |  | 1.000(0.986-1.014) | 0.960 | 15.504 | 0.114 |  | -0.0001 | 0.710 |
|  |  | weighted median |  | 0.998(0.993-1.003) | 0.515 |  |  |  |  |  |

Table S6 Causal effect of percentage of saturated fatty acids to total fatty acids on biliary tract calculus and inflammation

Table S7 Causal effect of saturated fatty acids on biliary tract calculus and inflammation

| Exposure | Outcome | MR method | Number of SNP | OR (95% CI) | *P* value | Test of heterogeneity | |  | Test of pleiotropy | |
| --- | --- | --- | --- | --- | --- | --- | --- | --- | --- | --- |
|  |  |  |  |  |  | Cochrane Q test | *P* of  heterogeneity |  | MR-Egger intercept | *P* of  pleiotropy |
| Saturated fatty acids | Calculus of bile duct without cholangitis or cholecystitis | Inverse variance weighted | 13 | 1.001(0.996-1.006) | 0.577 | 41.827 | 3.56e-05 |  |  |  |
|  |  | MR Egger |  | 1.019(0.957-1.086) | 0.551 | 40.627 | 2.79e-05 |  | -0.0004 | 0.580 |
|  |  | weighted median |  | 1.000(0.996-1.005) | 0.780 |  |  |  |  |  |
|  | Calculus of gallbladder without cholecystitis | Inverse variance weighted | 16 | 1.005(0.999-1.011) | 0.052 | 23.989 | 0.065 |  |  |  |
|  |  | MR Egger |  | 0.998(0.970-1.026) | 0.907 | 23.989 | 0.052 |  | 0.0002 | 0.604 |
|  |  | weighted median |  | 1.003(0.996-1.010) | 0.299 |  |  |  |  |  |
|  | Cholecystitis | Inverse variance weighted | 21 | 1.002(0.999-1.005) | 0.079 | 26.101 | 0.162 |  |  |  |
|  |  | MR Egger |  | 0.999(0.991-1.006) | 0.840 | 24.700 | 0.170 |  | 0.0001 | 0.312 |
|  |  | weighted median |  | 1.000(0.996-1.004) | 0.839 |  |  |  |  |  |
|  | Calculus of gallbladder with acute cholecystitis | Inverse variance weighted | 11 | 1.000(0.997-1.003) | 0.706 | 14.152 | 0.166 |  |  |  |
|  |  | MR Egger |  | 0.999(0.967-1.033) | 0.980 | 14.147 | 0.117 |  | 82.50e-05 | 0.955 |
|  |  | weighted median |  | 1.001(0.998-1.005) | 0.340 |  |  |  |  |  |

Table S8 Multiple testing correction: the adjusted *P* values of causal effects of exposures on outcomes

Abbreviations: pctMUFA, percentage of MUFAs to total FAs; pctPUFA, percentage of PUFAs to total FAs; PUTOMU, percentage of polyunsaturated fatty acids to monounsaturated fatty acids; pctSFA, percentage of saturated fatty acids to total FAs

| Outcomes | Exposures | | | | | | |
| --- | --- | --- | --- | --- | --- | --- | --- |
|  | MUFA | PUFA | pctMUFA | pctPUFA | PUTOMU | SFA | pctSFA |
| Calculus of bile duct without  cholangitis or cholecystitis | 0.7322424 | 0.5637366 | 0.7885275 | 0.5468407 | 1 | 0.8085722 | 0.9182168 |
| Calculus of gallbladder  without cholecystitis | 0.1382356 | 0.3349312 | 0.3481739 | 0.1876 | 0.1604496 | 0.1830457 | 0.3128186 |
| cholecystitis | 0.3244131 | 0.418533 | 0.1192678 | 0.1786324 | 0.1061634 | 0.1395102 | 0.3688632 |
| Calculus of gallbladder  with acute cholecystitis | 0.5336794 | 0.2321236 | 0.4014148 | 0.5312066 | 0.5677298 | 0.706839674 | - |

Table S9 Causal effects of fatty acids on cholecystitis based on FinnGen Consortium

| Outcome | Exposure | MR method | Number of SNP | OR (95% CI) | *P* value | Test of heterogeneity | |  | Test of pleiotropy | |
| --- | --- | --- | --- | --- | --- | --- | --- | --- | --- | --- |
|  |  |  |  |  |  | Cochrane Q test | *P* of  heterogeneity |  | MR-Egger intercept | *P* of  pleiotropy |
| Cholecystitis | MUFA | Inverse variance weighted | 12 | 0.852(0.524-1.382) | 0.517 | 9.798 | 0.548 |  |  |  |
|  |  | MR Egger |  | 0.829(0.242-2.844) | 0.773 | 9.796 | 0.458 |  | 0.0009 | 0.964 |
|  |  | weighted median |  | 0.797(0.398-1.593) | 0.521 |  |  |  |  |  |
|  | PUFA | Inverse variance weighted | 21 | 0.919(0.685-1.232) | 0.572 | 12.897 | 0.881 |  |  |  |
|  |  | MR Egger |  | 0.882(0.469-1.661) | 0.703 | 12.878 | 0.844 |  | 0.0019 | 0.889 |
|  |  | weighted median |  | 0.915(0.618-1.354) | 0.659 |  |  |  |  |  |
|  | pctMUFA | Inverse variance weighted | 28 | 1.12(0.929-1.349) | 0.232 | 30.25 | 0.303 |  |  |  |
|  |  | MR Egger |  | 1.243(0.95-1.626) | 0.123 | 29.002 | 0.31 |  | -0.0086 | 0.299 |
|  |  | weighted median |  | 1.177(0.951-1.456) | 0.133 |  |  |  |  |  |
|  | pctPUFA | Inverse variance weighted | 23 | 0.744(0.579-0.957) | 0.021 | 17.534 | 0.733 |  |  |  |
|  |  | MR Egger |  | 0.814(0.523-1.268) | 0.373 | 17.3 | 0.692 |  | -0.0049 | 0.634 |
|  |  | weighted median |  | 0.756(0.549-1.041) | 0.087 |  |  |  |  |  |
|  | PUTOMU | Inverse variance weighted | 30 | 0.854(0.685-1.064) | 0.161 | 42.589 | 0.049 |  |  |  |
|  |  | MR Egger |  | 0.954(0.674-1.351) | 0.795 | 41.613 | 0.047 |  | -0.008 | 0.424 |
|  |  | weighted median |  | 0.846(0.659-1.084) | 0.187 |  |  |  |  |  |
|  | SFA | Inverse variance weighted | 18 | 0.892(0.574-1.385) | 0.61 | 23.179 | 0.143 |  |  |  |
|  |  | MR Egger |  | 0.5(0.166-1.504) | 0.235 | 21.491 | 0.16 |  | 0.02 | 0.278 |
|  |  | weighted median |  | 0.651(0.371-1.14) | 0.133 |  |  |  |  |  |
|  | pctSFA | Inverse variance weighted | 8 | 0.647(0.349-1.2) | 0.167 | 9.121 | 0.244 |  |  |  |
|  |  | MR Egger |  | 0.484(0.035-6.633) | 0.606 | 9.045 | 0.171 |  | 0.01 | 0.829 |
|  |  | weighted median |  | 0.688(0.316-1.498) | 0.346 |  |  |  |  |  |

Abbreviations: pctMUFA, percentage of MUFAs to total FAs; pctPUFA, percentage of PUFAs to total FAs; PUTOMU, percentage of polyunsaturated fatty acids to monounsaturated fatty acids; pctSFA, percentage of saturated fatty acids to total FAs
