# Supplementary figures and images for: Associations of fatty acids with the risk of biliary tract calculus and inflammation: a Mendelian randomization study
Source: Lipids Health Dis. 2024 Jan 8;23:8. doi: 10.1186/s12944-023-01989-8 (PMC10773125; doi:10.1186/s12944-023-01989-8)

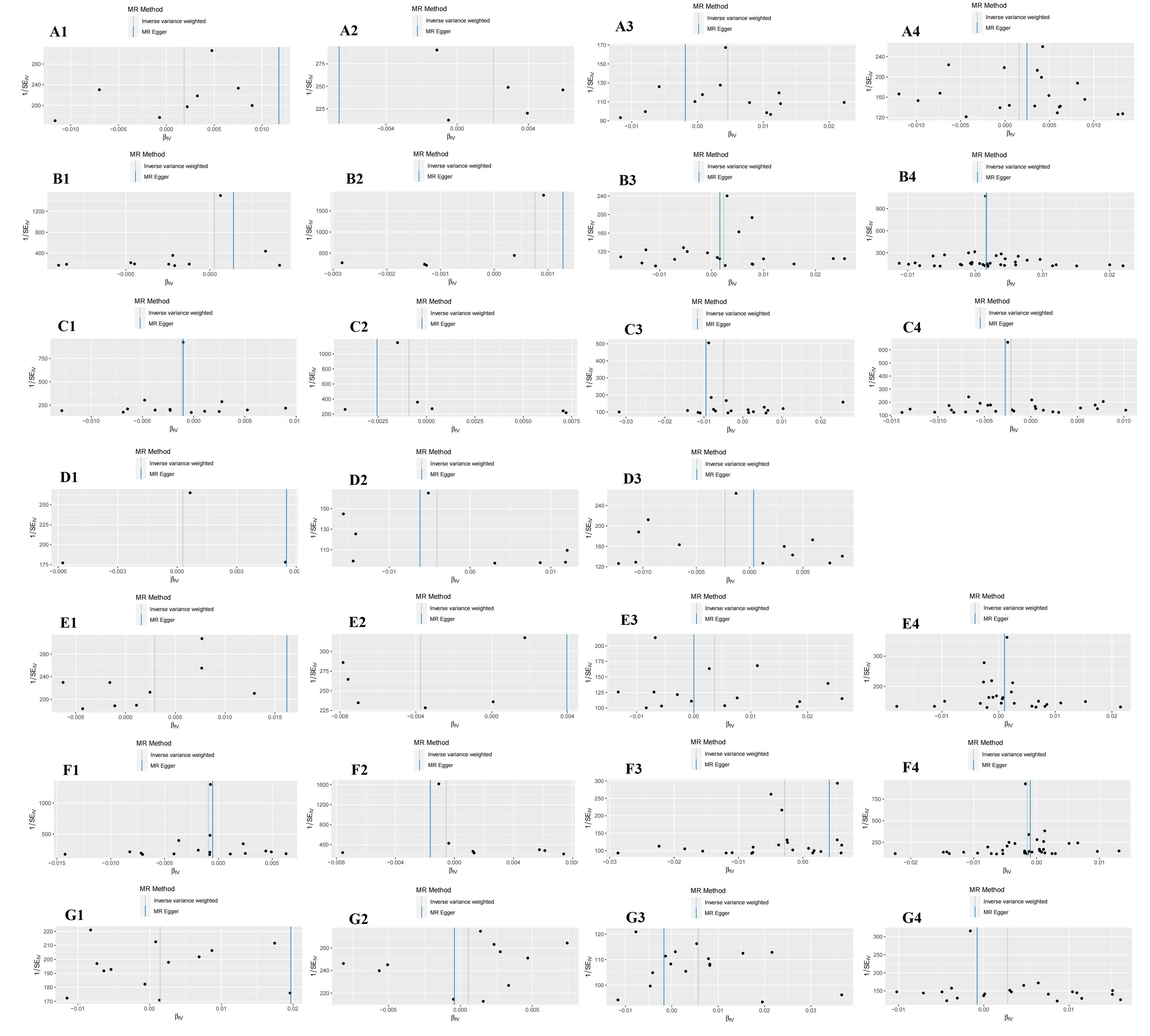

Supplement: Supplementary file 5 — Supplementary Figure2: Funnel plots of the enrolled SNPs. (A1-A4) MUFA-related SNPs, (B1-B4) Percentage of MUFA to total FAs-related SNPs, (C1-C4) Percentage of PUFA to total FAs-related SNPs, (E1-E4) PUFA-related SNPs, (F1-F4) Percentage of PUFA to MUFA-related SNPs, (G1-G4) SFA-related SNPs in calculus of bile duct without cholangitis or cholecystitis, calculus of gallbladder with acute cholecystitis, calculus of gallbladder without cholecystitis, and cholecystitis, respectively. (D1-D3) Percentage of SFA to total FAs-related SNPs in calculus of bile duct without cholangitis or cholecystitis, calculus of gallbladder without cholecystitis, and cholecystitis, respectively. [file 12944_2023_1989_MOESM5_ESM.jpg]

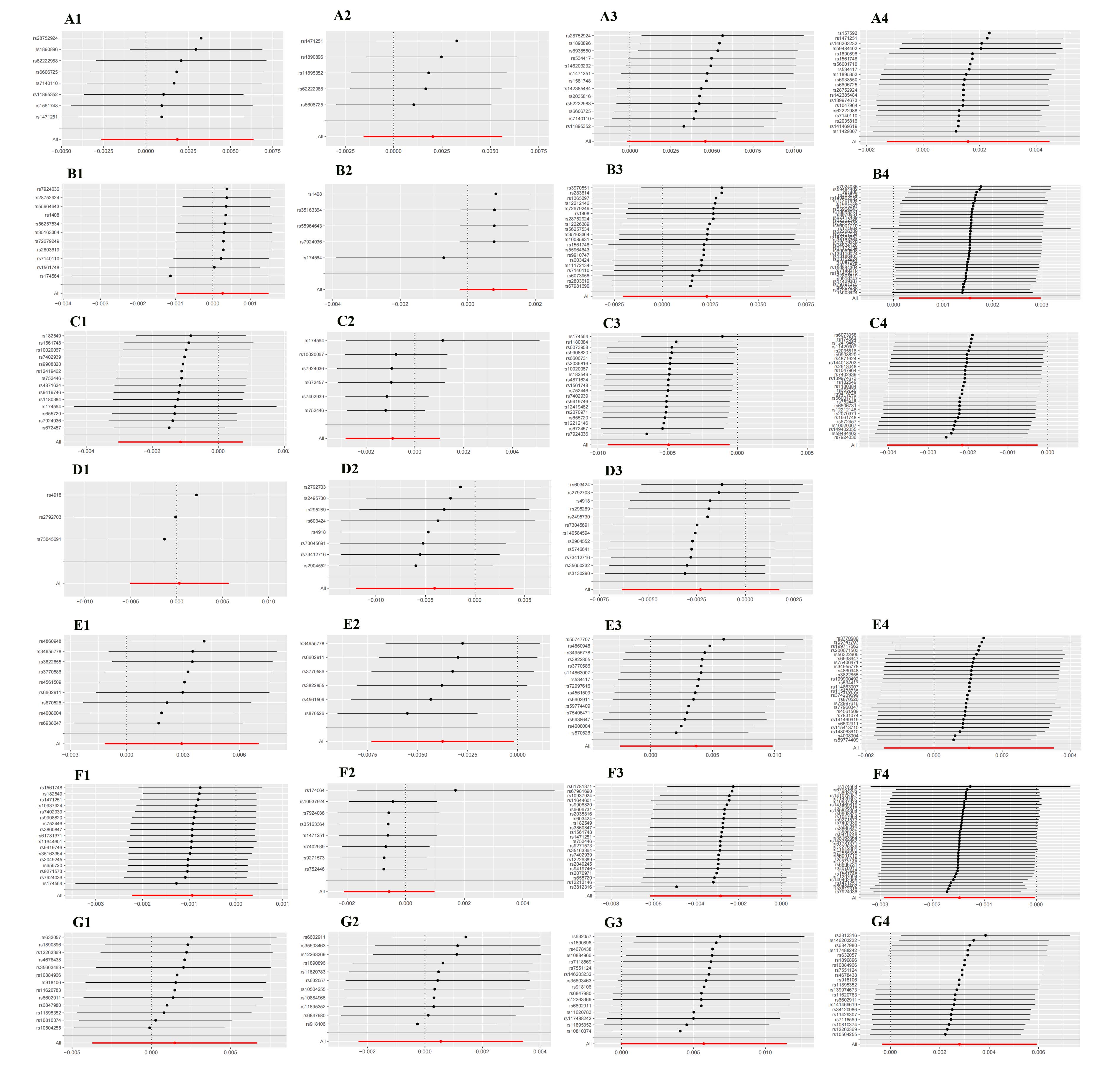

Supplement: Supplementary file 6 — Supplementary Figure3: The leave-one-out analysis. (A1-A4) MUFA-related SNPs, (B1-B4) Percentage of MUFA to total FAs-related SNPs, (C1-C4) Percentage of PUFA to total FAs-related SNPs, (E1-E4) PUFA-related SNPs, (F1-F4) Percentage of PUFA to MUFA-related SNPs, (G1-G4) SFA-related SNPs in calculus of bile duct without cholangitis or cholecystitis, calculus of gallbladder with acute cholecystitis, calculus of gallbladder without cholecystitis, and cholecystitis, respectively. (D1-D3) Percentage of SFA to total FAs-related SNPs in calculus of bile duct without cholangitis or cholecystitis, calculus of gallbladder without cholecystitis, and cholecystitis, respectively. [file 12944_2023_1989_MOESM6_ESM.jpg]
